# USE OF ARTIFICIAL INTELLIGENCE FOR THE EVALUATION OF THE ENTRANCE EXAMINATION OF THE BRAZILIAN SOCIETY OF SHOULDER AND ELBOW SURGERY

**DOI:** 10.1590/1413-785220263404e300345

**Published:** 2026-07-17

**Authors:** Caio Henrique Kenchian, Karolina Stephany Pereira Ferreira, Lucas Pereira Sarmento, Marcelo Silveira Teixeira, Renato Aroca Zan, Marcel Jun Sugawara Tamaoki

**Affiliations:** 1 Universidade Federal de Sao Paulo, Escola Paulista de Medicina, Departamento de Ortopedia e Traumatologia, Sao Paulo, SP, Brazil.

**Keywords:** Educational Measurement, Education, Medical, Artificial Intelligence, Orthopedics, Natural Language Processing, Avaliação Educacional, Educação Médica, Inteligência Artificial, Ortopedia, Processamento de Linguagem Natural

## Abstract

**Introduction:**

AI is increasingly used for medical education and assessment, yet effectiveness on specialized exams remains underexplored. We aim to compare multiple AI models with national human averages on SBCOC entrance exams (2021–2023), evaluate answer accuracy and citation reliability, and contrast models.

**Methods:**

Five models—ChatGPT-4 (standard and literature-trained), ChatGPT-o1-pro, Gemini, and Meta Llama 3.1—answered official SBCOC exams (50 items/year) individually using a unified prompt and identical images. Each response included one choice (A–D) and a cited source. Outcomes were accuracy and source reliability (peer-reviewed articles/textbooks versus websites). Statistics used chi-square tests and one-way ANOVA with Tukey post-hoc (p<0.05).

**Results:**

Across 150 questions, ChatGPT-o1-pro led (66%, 62%, 68%) and exceeded human national averages each year (p<0.05). Gemini (40%, 28%, 38%) and Meta Llama 3.1 (32%, 44%, 50%) underperformed relative to humans, while both ChatGPT-4 versions hovered near the ≥50% pass threshold without significant differences. Regarding sources, ChatGPT-o1-pro and Llama predominantly cited articles or books, ChatGPT-4 alternated between literature and websites, and Gemini did not specify references.

**Conclusions:**

ChatGPT-o1-pro outperformed the national human average and mostly used credible sources; ChatGPT-4 matched humans, while Gemini and Meta Llama 3.1 lagged. ***Level of evidence IV; Case series.***

## RESUMO

**Introdução:**

A IA é cada vez mais usada na educação e avaliação médicas, mas sua efetividade em exames especializados permanece pouco explorada. O objetivo foi comparar modelos de IA às médias humanas nos exames de ingresso da SBCOC (2021–2023), avaliar acurácia e confiabilidade das citações e contrastar modelos.

**Métodos:**

Cinco modelos—ChatGPT-4 (padrão e treinado com literatura), ChatGPT-o1-pro, Gemini e Meta Llama 3.1—responderam às provas da SBCOC (50 itens/ano) individualmente, com prompt unificado e imagens idênticas. Cada resposta continha uma alternativa (A–D) e uma fonte.

**Desfechos:**

acurácia e confiabilidade das fontes (artigos/livros vs. sites). Estatística: qui-quadrado e ANOVA de uma via com pós-teste de Tukey (p<0,05).

**Resultados:**

Em 150 questões, o ChatGPT-o1-pro liderou (66%, 62%, 68%) e superou médias humanas em todos os anos (p<0,05). Gemini (40%, 28%, 38%) e Meta Llama 3.1 (32%, 44%, 50%) abaixo dos humanos; ambas as versões do ChatGPT-4 próximas ao limiar de aprovação ≥50%, sem diferenças significativas. Quanto às fontes, ChatGPT-o1-pro e Llama citaram predominantemente artigos/livros; o ChatGPT-4 alternou entre literatura e sites; Gemini não especificou referências.

**Conclusões:**

O ChatGPT-o1-pro superou a média humana e usou majoritariamente fontes confiáveis; o ChatGPT-4 igualou os humanos; Gemini e Meta Llama 3.1 ficaram aquém. ***Nível de evidência IV; série de casos.***

## INTRODUCTION

Artificial intelligence (AI) has undergone remarkable evolution since its early applications in the 1950s, when it was limited to logic-based and symbolic reasoning systems, to the complex machine learning models we know today. In the field of medicine, AI began to gain relevance with the development of specialized systems, such as MYCIN in the 1970s, one of the first to assist in the diagnosis of bacterial infections^1^. However, it was never used clinically due to technical and trust limitations^2^. Since then, AI techniques have evolved considerably, driven by improvements in processing power and the emergence of deep neural networks, enabling AI to perform tasks that once seemed unattainable.

Today, AI is successfully applied in various medical specialties^3–5^. However, not all attempts to integrate AI into medicine have been successful. Many systems struggle to handle the complexity and variability of real-world clinical conditions^2^. Additionally, the lack of high-quality data and the resistance of healthcare professionals to fully adopt these technologies remain recurring barriers.

In contrast, in the field of medical education, AI adoption has been more widely accepted^6^. Tools like ChatGPT provide support to students, especially in simulation scenarios and problem-based tutorials, offering a more immersive and personalized learning experience^7^. However, much of the information provided by these tools can be inaccurate or sometimes inappropriate. Therefore, these advancements need to be continuously evaluated to ensure that AI is used safely and effectively in medical practice.

Natural language processing (NLP) models, particularly large language models (LLMs) like ChatGPT, have made significant advancements in recent years. Various studies^8–10^ have already explored these models, demonstrating not only their high capability in solving medical exams but also highlighting the superiority of newer models.

In addition to ChatGPT, other NLP models have also gained relevance in medicine. Gemini, developed by Google DeepMind, combines language processing with the analysis of large volumes of clinical data to provide more accurate diagnoses and personalized treatments^11^. It is important to note that this study employed the standard publicly available Gemini model, not the medically specialized ‘Med-Gemini’ described by Saab et al^11^. These models stand out due to their unique architectures and learning capabilities; however, it is essential to continuously monitor their accuracy and safety to ensure their ethical and secure use.

The use of AI in medical examinations also raises concerns about its reliability and accuracy. Although natural language models have shown promising performance, a systematic evaluation is necessary to ensure that the results are consistent and accurate^12^. Studies validating the effectiveness of these models in clinical simulations and across different medical specialties are essential to ensure that these technologies effectively contribute to the training of future specialists^13^.

Therefore, although the advancements are remarkable, there are still important gaps to be addressed. More detailed studies are needed to assess AI performance in image interpretation and ensure the accuracy and robustness of the information provided. These are areas that require improvement for AI to be safely and effectively integrated into both medical education and clinical practice.

The justification for this study lies in the increasing use of artificial intelligence (AI) as a support tool in medical education and clinical practice. Although AI models such as ChatGPT, Gemini, and Meta Llama demonstrate great potential in solving complex problems, there is a gap in evaluating their performance compared to human candidates, especially in more specialized exams like those of the Brazilian Society of Shoulder and Elbow Surgery (SBCOC).

Thus, this study aims to compare the performance of AI models among themselves and in comparison to the national averages of human candidates, both in terms of response accuracy and the reliability of the sources used, in the entrance exams of the Brazilian Society of Shoulder and Elbow Surgery (SBCOC) from 2021 to 2023. It will also compare trained versions, with access to medical literature used as a reference, to untrained versions, with free access to the internet. Additionally, the evaluation of the AIs will include questions that involve image interpretation for resolution.

## METHODS

### Study Design and Overview

The evaluation of different Artificial Intelligence (AI) models was conducted using the official examinations of the Brazilian Society of Shoulder and Elbow Surgery (SBCOC) from 2021, 2022, and 2023. These admission examinations comprise fifty multiple-choice questions with four alternatives, only one of which is correct, and the SBCOC supplied the official exams, corresponding answer keys, and anonymized statistical data on candidate performance. Five AI models were evaluated—ChatGPT-4 in two modalities (a standard version with open internet access and an isolated version restricted exclusively to the exam's reference literature), ChatGPT-o1-pro, Gemini (public version, without the medical enhancements of Med-Gemini), and Meta Llama 3.1—enabling direct comparison between models with unrestricted access to external information and those limited to a closed reference set.

To standardize administration, a single prompt was used across all models: "I will provide a multiple-choice question on the topic of shoulder and elbow surgery, taken from an examination for orthopedic surgeons. I want you to select the best alternative to answer the question, using the information provided in the question as well as data from your database or the internet. I also want you to provide the source used to solve the question. The answer must be in the format of one alternative: A, B, C, or D".

Each question was presented individually, because administering the entire exam at once caused oversimplification and higher error rates; when questions included images, the same images were attached within each AI's interface to ensure identical visual content. Model responses were assessed on two criteria: (1) accuracy, via direct comparison with the official SBCOC answer key; and (2) source usage, analyzing the reliability of cited references, with textbooks and peer-reviewed articles deemed reliable and websites or non-validated materials deemed unreliable.

Tests were conducted under standardized conditions: all models received identical instructions and were evaluated on their native user platforms within a short time frame to minimize performance variations from back-end updates, thus promoting consistency and comparability across models.

The collected data underwent statistical analysis with the objectives of comparing performance among AI models and against human performance (national average of SBCOC candidates across the three years) and assessing the reliability of cited sources, thereby ensuring methodological rigor for both answer-accuracy comparisons and critical appraisal of reference quality.

### Statistical Analysis

To evaluate significant differences in AI performance among models and compared to human candidates across the study period (2021–2023), chi-square tests (χ^2^) were applied to categorical variables (correct vs. incorrect answers), with a significance level set at p<0.05. Additionally, an analysis of variance (ANOVA) followed by Tukey's multiple comparison tests was used to compare average performance scores among the AI models. The tested hypothesis was that significant differences would exist among the models and between the different exam years. All statistical analyses were conducted using JASP software, version 0.19.2 (2024), with a significance threshold of 0.05.

### Ethical Considerations

This study was conducted in accordance with the ethical principles. It was approved by the Research Ethics Committee of the Federal University of São Paulo – Escola Paulista de Medicina (UNIFESP - EPM) and registered on the "Plataforma Brasil" under the CAAE number 81368024.9.0000.5505. The research involved the analysis of data from candidates who took the entrance exam of the Brazilian Society of Shoulder and Elbow Surgery. All data were anonymized prior to analysis to ensure the confidentiality and privacy of the participants. No identifiable personal information was used at any stage of the study.

## RESULTS

The analyses were conducted under two circumstances. The first analysis employed the chi-square (χ^2^) contingency test, which evaluates deviations in proportions and assesses differences in the observed proportions of correct answers among AI models compared to the established pass threshold of 50% (Table 1). The chi-square test identified significant differences among the AI models across the analyzed years (χ^2^; p<0.05). In 2021, ChatGPT-o1-pro had the highest accuracy rate (66%), followed by ChatGPT-4 (60%), while Gemini (40%) and Meta Llama 3.1 (32%) performed worse and did not reach the passing threshold. In 2022, ChatGPT-o1-pro maintained the best performance (62%), while ChatGPT-4 (50%) and Meta Llama 3.1 (44%) had intermediate performance, and Gemini had the lowest score (28%). In 2023, ChatGPT-o1-pro again achieved the highest accuracy rate (68%), being the only AI to consistently outperform the others over the years.

**Table 1:** Percentage of correct answers among five different artificial intelligence models.

Year	AI Model	Correct Answers*	Correct	Passed	χ^2^ (p-value)
2021	GPT-4	30	60%	Yes	>11.07 (p=0.03)
	GPT-4 (trained)	26	52%	Yes	
	GPT-o1-pro	33	66%	Yes	
	Gemini	20	40%	No	
	Meta Llama 3.1	16	32%	No	
2022	GPT-4	25	50%	Yes	>9.48 (p=0.01)
	GPT-4 (trained)	28	56%	Yes	
	GPT-o1-pro	31	62%	Yes	
	Gemini	14	28%	No	
	Meta Llama 3.1	22	44%	No	
2023	GPT-4	24	48%	No	>7.79 (p=0.01)
	GPT-4 (trained)	26	52%	Yes	
	GPT-o1-pro	34	68%	Yes	
	Gemini	19	38%	No	
	Meta Llama 3.1	25	50%	Yes	

* The calculated χ^2^ value (df = 5) exceeded the critical value throughout the period, indicating a significant difference at the 5% level regarding the proportions of correct answers. Therefore, the null hypothesis (H_o_), which suggested no differentiation between the AIs, is rejected.

The second analysis, focusing on individual model performances, was conducted using a one-way ANOVA without repetition, performed after a positive parametricity test, to verify whether there were differences in the geometric mean accuracy rates among the different AI models, including accuracy in text-based questions and image-based questions (Table 2). The geometric mean was selected due to the percentage-based nature of the data, as it is particularly suitable for averaging proportional rates or indices and mitigates the disproportionate impact of extreme values. Table 2 shows that ChatGPT-o1-pro achieved the highest average performance (65.3%), with a significant difference confirmed by ANOVA (p=0.03). In image-based questions, all AIs except Gemini had an average performance above 50%, with ChatGPT-o1-pro standing out (73.7%), being the only model to consistently exceed 50% accuracy in each individual exam (Table 3).

**Table 2:** Analysis of variance between the average correct answers for text questions between 2021 and 2023.

AI Model	Average Correct Answers (2021–2023) and 95% Confidence Interval*	ANOVA**
GPT-4	26.2 (24.2 - 27.8)	F > 6553 (p=0.03)
GPT-4 (trained)	26.6 (27.2 - 28.0)	
GPT-o1-pro	32.6 (29.8 - 37.3)	
Gemini	17.0 (15.2 - 18.0)	
Meta Llama 3.1	21.2 (19.2 - 23.2)	

* The confidence intervals for percentages were calculated using the standard error (SE) corrected by stratification: √p(1-p)/n, and with 95% confidence = p’ ± 1.96 × SE.

** The ANOVA test revealed a significant difference between the AIs, confirmed by the Tukey post hoc test, which showed differences in the means, particularly with the GPT-o1-pro.

**Table 3:** Analysis of variance between the average correct answers for image questions between 2021 and 2023.

AI Model	Average* Correct Answers (2021–2023) and 95% Confidence Interval**	ANOVA***
GPT-4	2.0 (0.8 - 2.8)	F > 6553 (p=0.03)
GPT-4 (trained)	2.0 (1.0 - 3.0)	
GPT-o1-pro	2.6 (2.1 - 3.1)	
Gemini	1.0 (0.8 - 1.8)	
Meta Llama 3.1	2.0 (1.2 - 2.8)	

* Average of 3.66 image-based questions per exam/year.

** The confidence intervals for percentages were calculated using the standard error (SE) corrected by stratification: √p(1-p)/n, and with 95% confidence = p’ ± 1.96 × SE.

*** The ANOVA test revealed a significant difference between the AIs, confirmed by the Tukey post hoc test, which showed differences in the means, particularly with the GPT-o1-pro.

An inference was also made comparing candidates versus AIs, based on the percentage difference in accuracy rates, analyzed using the chi-square contingency test (Table 4). It is evident that ChatGPT-o1-pro consistently outperformed the national average of candidates in all years, being 14% superior in 2021 (p=0.04), 20% superior in 2022 (p=0.03), and 36% superior in 2023 (p=0.029). Meanwhile, Gemini and Meta Llama 3.1 were outperformed by the candidates in all years. ChatGPT-4, in both versions (trained and untrained), demonstrated statistically similar performance to the candidates.

**Table 4:** Percentage difference between candidates’ and AI's correct answers from 2021 to 2023.

Year	AI Model	Correct Answers	χ^2^ (p-value)
2021	GPT-4	30	Performance 3.3% better for AI** (p=0.07)
	GPT-4 (trained)	26	Performance 11.5% better for candidates (p=0.06)
	GPT-o1-pro*	33	Performance 14% better for AI** (p=0.04)
	Gemini	20	Performance 45% better for candidates (p<0.01)
	Meta Llama 3.1	16	Performance 82% better for candidates (p<0.01)
	Students	29	-
2022	GPT-4	25	Performance 4% better for candidates (p=0.07)
	GPT-4 (trained)	28	Performance 7.2% better for AI** (p=0.07)
	GPT-o1-pro*	31	Performance 20% better for AI** (p=0.03)
	Gemini	14	Performance 86% better for candidates (p<0.01)
	Meta Llama 3.1	22	Performance 18% better for candidates (p<0.01)
	Students	26	-
2023	GPT-4	24	Performance 4.7% better for candidates (p=0.07)
	GPT-4 (trained)	26	Performance 4% better for AI** (p=0.06)
	GPT-o1-pro*	34	Performance 36% better for AI** (p=0.03)
	Gemini	19	Performance 31.7% better for candidates (p=0.02)
	Meta Llama 3.1	25	Similar performance
	Students	25	-

* The average number of correct answers for the AI GPT-o1-pro over the three years was 32.6 (65.2%), showing a significant difference (p<0.05, as assessed by χ^2^) compared to the students› average of 26.6 (53%).

** The χ^2^ test did not reveal a significant difference between the AIs and candidates, despite GPT-5 and GPT-5 Trained having better percentage performances. Candidates outperformed only with Gemini and Meta Llama 3.1 (p<0.01 in comparison to both, as assessed by χ^2^).

Finally, as contributory elements for confirming the hypotheses of this study, an analysis was conducted to compare the bibliographic sources used by the AIs and their performance (Table 5). It was observed that ChatGPT-o1-pro and Meta Llama 3.1 exclusively used books and scientific articles across all three years. ChatGPT-4, on the other hand, alternated between websites and scientific literature, with an increase in the use of articles in 2023 (58%). The Gemini model did not specify the sources used, merely stating that it relied on articles, websites, and the internet in general, which were considered unreliable sources. The chi-square test (χ^2^) indicated that the preference for sources varied significantly among the AIs.

**Table 5:** Percentage difference in the bibliographic sources used by different AIs to solve the questions.

Year	AI Model	Books and Articles	Websites	χ^2^ (p-value) *
2021	GPT-4	23 (46%)	27 (54%)	p=0.03 (websites)
	GPT-o1-pro	50 (100%)	-	-
	Meta Llama 3.1	50 (100%)	-	-
2022	GPT-4	14 (28%)	36 (72%)	p=0.02 (websites)
	GPT-o1-pro	50 (100%)	-	-
	Meta Llama 3.1	50 (100%)	-	-
2023	GPT-4	29 (58%)	21 (42%)	p=0.04 (articles)
	GPT-o1-pro	50 (100%)	-	-
	Meta Llama 3.1	50 (100%)	-	-

* The calculated χ^2^ value (df = 2) exceeded the tabled value.

## DISCUSSION

### Principal Results

This study evaluated AI performance on SBCOC medical exams (2021–2023) against the national average of human candidates, assessing answer accuracy and the use of reliable bibliographic sources. Regarding the passing threshold (≥50% correct answers), both trained ChatGPT-4 and ChatGPT-o1-pro met the criterion across all years; between them, ChatGPT-o1-pro outperformed the candidates and achieved the highest average number of correct answers, including on image-based questions, standing out particularly in 2022 and 2023 when candidate performance declined markedly. A model-specific analysis revealed a performance gap: Gemini and Meta Llama 3.1 obtained the lowest results relative to other AIs and to humans, while trained and standard ChatGPT-4 showed no significant difference from each other and did not differ significantly from the candidates.

Source behavior further differentiated models: ChatGPT-4 frequently relied on websites (a limitation due to lower reliability), ChatGPT-o1-pro consistently relied on trustworthy sources, and Gemini did not specify sources—citing only "articles, websites, and the internet in general"—hindering quality appraisal. Notably, although Meta Llama 3.1 performed worse than other AIs, it referred exclusively to ostensibly reliable sources; however, its author–book pairings were often inaccurate, indicating fabricated attributions and underscoring the need to carefully analyze AI-generated responses for invented information and references.

### Study Limitations

Although the testing was conducted within a close time frame and on each model's standard usage platform to minimize system-related variability, the possibility of performance fluctuations due to back-end factors cannot be fully excluded. Updates to infrastructure, adjustments in model parameters, or system-level optimizations may have occurred during the evaluation period, introducing uncontrolled variation into the results. While efforts were made to standardize the conditions of application, these back-end variations remain an inherent limitation in the assessment of proprietary AI models, as researchers have no direct control or visibility over the dynamic changes that may take place in their underlying architectures.

### Comparison with Prior Work

This review situates the present study within a growing literature showing strong AI performance on medical multiple-choice examinations and clarifies key limitations and contributions.

Prior work on the United States Medical Licensing Examination (USMLE)^10^ reported that ChatGPT correctly answered more than 60% of questions, reaching the passing threshold for Steps 1 and 2 and suggesting knowledge comparable to a third-year medical student.

On the Orthopaedic In-Training Examination (OITE)^8^, ChatGPT-3.5 answered 196 of 360 questions (54.3%), whereas ChatGPT-4 achieved 265 of 360 (73.6%)—equivalent to the performance of a fifth-year resident (PGY-5) and above the minimum passing score for Part I of the American Board of Orthopaedic Surgery Exam; notably, ChatGPT-4 provided verifiable sources in 87.9% of responses, versus 47.2% for the earlier version.

ChatGPT-4 also performed strongly on the Neurological Surgeons Self-Assessment Neurosurgery Exam (SANS)^9^, attaining high accuracy across question categories (tumors, cerebrovascular conditions, trauma, pediatrics) and outperforming medical students, neurosurgery residents, and the national average of SANS users, implying it would likely pass the American Board of Neurological Surgery primary examination.

Additionally, Costa et al. reported that ChatGPT-4 answered 61.05% of questions correctly on the 2022 TEOT first-phase exam—surpassing the ≥60% passing threshold—reinforcing that LLMs can meet Brazilian specialist-exam benchmarks in orthopedics^14^.

This article makes a distinct contribution by benchmarking multiple AI models (ChatGPT-4, ChatGPT-o1-pro, Gemini, Meta Llama 3.1, and a trained ChatGPT-4) against national human averages on SBCOC entrance exams (2021–2023), assessing not only overall accuracy but also the reliability of cited sources within a highly specialized surgical domain. The findings align with and extend prior literature by demonstrating model behavior in a narrower, domain-specific context, reinforcing the potential of advanced systems—particularly ChatGPT-o1-pro—as supplementary educational tools that aid preparation for specialized examinations and enhance learning outcomes, while underscoring the need for ongoing critical monitoring of AI-generated content and sources and for continuous refinement to ensure safe, effective use in educational and clinical settings.

## CONCLUSION

The average performance of ChatGPT-o1-pro consistently exceeded the national average of human candidates, and it predominantly cited reliable sources; by contrast, ChatGPT-4 (across all versions) demonstrated average performance comparable to that of humans and consistent across versions, whereas Gemini and Meta Llama 3.1 exhibited lower average performance. Persistent challenges include the rigorous validation of bibliographic sources provided by AI models, which necessitates ongoing evaluation and cautious deployment, particularly in medical education and clinical practice.

## Data Availability

The content underlying the research text is contained in the manuscript.
